# Antidiabetic and Antioxidant Effects and Phytochemicals of Mulberry Fruit (*Morus alba* L.) Polyphenol Enhanced Extract

**DOI:** 10.1371/journal.pone.0071144

**Published:** 2013-07-30

**Authors:** Yihai Wang, Limin Xiang, Chunhua Wang, Chao Tang, Xiangjiu He

**Affiliations:** School of Pharmaceutical Sciences, Wuhan University, Wuhan, China; University of Sassari, Italy

## Abstract

The antidiabetic and antioxidant activities of the ethyl acetate-soluble extract (MFE) of mulberry fruit *(Morus alba* L.) were investigated. *In vitro*, MFE showed potent α-glucosidase inhibitory activity and radical-scavenging activities against DPPH and superoxide anion radicals. *In vivo*, MFE could significantly decrease fasting blood glucose (FBG) and glycosylated serum protein (GSP), and increase antioxidant enzymatic activities (SOD, CAT, GSH-Px) in streptozotocin (STZ)-induced diabetic mice. Bioactivity-guided fractionation of the MFE led to the isolation of 25 phenolic compounds, and their structures were identified on the basis of MS and NMR data. All the 25 compounds were isolated from mulberry fruit for the first time. Also, the α-glucosidase inhibitory activity and antioxidant activity of the phenolics were evaluated. Potent α-glucosidase inhibitory and radical-scavenging activities of these phenolics suggested that they may be partially responsible for the antidiabetic and antioxidant activities of mulberry fruit.

## Introduction

Diabetes mellitus, which is characterized as a consequence of genetically based predisposition and dietary indiscretion, is a chronic metabolic disease with the highest rates of prevalence and mortality in both developed and developing countries. It has been reported to associate with oxidative damage although the leading mechanism of diabetic complications remains unclear [1]–[3]. Prevention of oxidative damage with natural antioxidants and control of postprandial hyperglycemia, by inhibiting digestive enzymes such as α-glucosidase, a main glycosidase hydrolases found on the luminal surface of enterocytes, are two important diabetic prevention strategies. Phenolic phytochemical which function as chemopreventive agents against oxidative damage have attracted growing public attention. Consumption of fruits and vegetables rich of phenolics has been linked to the decreasing risk of developing chronic diseases by the reduction of oxidative stress and inhibition of macromolecular oxidation [4], [5]. From this point of view, intensive efforts have been made to search for more effective and safe antioxidants and α-glucosidase inhibitors in natural materials to develop physiological and functional food for the prevention and treatment of diabetes [6]–[8].

Mulberries (*M. alba* L.) are large, deciduous trees native to warm, temperate, and subtropical regions of Asia, Africa, and the Americas. Mulberry fruits are delicious, fleshy, succulent berries, which are low in calories and contain health promoting phytonutrient compounds like polyphenols, minerals and vitamins that are essential for optimum health. It has long been used to treat and prevent diabetes, and as a general tonic to enhance health in traditional oriental medicine. Consumption of mulberry fruit has been linked to the prevention of various chronic diseases. Mulberry extract has been reported to have potent antioxidant activity [9], [10], antitumor activity [11], [12], hypolipidemic effect [10], [13], [14], macrophage activating effect [15], and neuroprotective activity [16], [17]. Many of these bioactivities were linked to the presence of phenolics in mulberry fruit. Like other berry fruit, mulberry fruit contains high amounts of flavonoids, including anthocyanins that are responsible for its color, and other phenolics. Anthocyanins extracted from mulberry has been reported to exhibit potent neuroprotective [16] and antitumor activity [11]. Also, some alkaloids were identified from mulberry fruit and show strong bioactivities [15], [18].

Despite the various biological activities of mulberry fruit extracts, the bioactive constituents responsible for its antidiabetic and antioxidant activities are not clear yet. In the present research, the hypoglycemic and antioxidant abilities of the polyphenol-rich part of mulberry fruit were evaluated *in vitro* and *in vivo*. Bioactivity-guided fractionation was used to investigate the phytochemical of mulberry fruit polyphenol-rich extract, and to isolate and identify the bioactive compounds with potent hypoglycemic and antioxidant activities.

## Materials and Methods

### Ethics Statement

All protocols involving animal experiments were approved by the ethics committee of Wuhan University (Wuhan, China). The mice were given 5% glucose solution orally in order to stave off the hypoglycemia during the first day after the injection of STZ. At the end of the experiments, they were sacrificed under diethyl ether anesthesia, and all efforts were made to minimize suffering.

### Plant Material

Dried mulberry fruit (*M. alba* L.) were purchased from Wuhan Qiangkang Pharmaceutical Co. Ltd. (Wuhan, China).

### Chemicals and Reagents

α-Glucosidase type I (EC 3.2.1.20) from baker's yeast, p-nitrophenyl α-D-glucopyranoside (PNPG), streptozotocin (STZ), were purchased from Sigma-Aldrich Chemical Co. (St. Louis, MO). Acarbose tablets were purchased from Bayer Health Care Company Ltd. (Beijing, China). Phenazine methosulfate was purchased from J&K Chemical Ltd. (Shanghai, China). 2,2-Diphenyl-1-picrylhdrazyl (DPPH) was product of Aladdin Chemistry Co. Ltd. (Shanghai, China). Silica gel (200–300 mesh, Anhui Liangchen Silicon Material Co. Ltd. Anhui, China), Sephadex LH-20 (Pharmacia Biothech AB, Uppsala, Sweden), and ODS (40–60 *μ*m, Merck KGaA, Darastadt, Germany) were used for column chromatography. HPLC-grade methanol was purchased from Jiangsu Hanbang Science and Technology Co. Ltd. (Nanjing, China). All other analytical chemicals and reagents were purchased from Sinopharm Chemical Regent Co. Ltd. (Shanghai, China).

### Instrumentation

All NMR spectra were obtained on a Bruker DPX-400 spectrometer using standard Bruker pulse programs (Bruker BioSpin GmbH, Rheinstetten, Germany). ESI-MS data were obtained on an Agilent 6120 series MS system (Agilent Technologies, Inc., Santa Clara). HPLC analysis was performed on a Waters (Milford, MA, USA) chromatographic system using an analytical ODS column (Amethyst C18-H, 4.6×250 mm, 5 *μ*m, Sepax Technologies Inc., Newark, NJ). A Waters 600 pump model equipped with a Waters 996 photodiode array detector and a Waters in-line degasser connected to Empower PDA software for data acquisition. Semi-preparative HPLC was carried out on a Rainin HPLC system composed of a Dynamax model HPXL solvent delivery system equipped with a Dynamax absorbance detector model UV-D II with the observing wavelength set at 205 nm (Rainin Instrument Co. Inc., Woburn, MA), and a semi-preparative ODS column (Cosmosil 5C18-MS- II, 10ID×250 mm, Nacalai Tesque, Kyoto, Japan) was used. Microplate reader was product of Kehua Technologies, Inc. (Shanghai, China).

### Preparation of Plant Extracts for Chemical and Bioactive Studies

Dried mulberry fruit (1.0 kg) was extracted three times using 3 volumes of 70% ethanol for 2 h at reflux. The filtrate was concentrated under vacuum at 50°C to afford 70% ethanol extract. The 70% ethanol extract was suspended in 1000 mL of distilled water and successively partitioned three times with the same volume of hexane, CHCl_3_, EtOAc, and n-butanol, which yielded hexane (4.73 g), CHCl_3_ (18.90 g), EtOAc (MFE: 4.65 g), n-BuOH (33.95 g) and water soluble extracts respectively, after removal of organic solvent under vacuum at 50°C. The extracts were stored at −20°C until used.

### Quantification of Total Phenolic and Flavonoid Content

The total phenolic content of the extracts was measured using the Folin-Ciocalteu colorimetric method modified by Wolfe *et al* [19]. Total phenolic contents of the extracts were expressed as milligrams of gallic acid equivalents per gram of extract (mg of GAE/g of extract) through the calibration curve with gallic acid. Data were reported as mean ± SD for triplicate measurements.

The total flavonoid content was determined according to a previously described method [20]. Total flavonoid contents of extracts were expressed as milligrams of rutin equivalents per gram of extract (mg of RE/g of extract) through the calibration curve with rutin. Data were reported as mean ± SD for triplicate measurements.

### Determination of DPPH Radical-scavenging Activity

The DPPH radical-scavenging assay was performed according to a previously described method [21]. All tests were performed in triplicate. Ascorbic acid and gallic acid were used as positive controls.

### Determination of Superoxide Anion Radical-scavenging Activity

Superoxide radical were generated by the NADH/PMS system according to a reported protocol [22]. Gallic acid was used as positive control. The data were expressed as EC_50_, which was defined as the final concentration of the tested sample required for the scavenging of superoxide anion radical by 50%.

### α-Glucosidase Inhibition Assay

The α-glucosidase inhibitory studies were performed spectrophotometrically on 96-well microplate reader according to a reported method [23]. Acarbose was used as positive control. The assay was performed in triplicate. The results were expressed as the sample concentration required to inhibit 50% of the enzyme activity (IC_50_).

### Animals

Male Kunming mice (18-20 g) and standard laboratory diet were purchased from the Laboratory Animal Center of Wuhan University. They were housed in an air-conditioned room with a 12 h light/dark cycle at temperature of 25±2°C and free access to food and tap water. All mice were adapted to the new environment for 3 days before the experiment.

### STZ-induced Hyperglycemia in Mice for *in vivo* Assay

Hyperglycemia was induced in overnight-fasted mice by intraperitoneal injection of 150 mg/kg body weight streptozotocin (STZ) using a 1% solution of freshly prepared STZ in 0.1 M citrate buffer (pH 4.5). The mice were given 5% glucose solution orally in order to stave off the hypoglycemia during the first day after the injection of STZ [24]. In testing the FBG of the mice, a standard protocol developed by the American National Institute of Health was carried out. Blood samples were drawn from the tail vein of the mice at 1 p.m. after a short fast from 7 a.m. the third day after STZ administration. The FBG was measured using the Accu-Check Active Blood Glucose Meter (Roche Diagnostics, Mannheim, Germany). Mice with FBG values >10 mmol/L were considered to be hyperglycemic.

Total 32 hyperglycemic mice were divided into four groups according to their FBG (8 mice per group): control (distilled water), MFE 100 (100 mg/kg BW), MFE 200 (200 mg/kg BW), and metformin (300 mg/kg BW). Sample suspended in distilled water containing 0.3% CMC-Na was administered *p.o.* two times a day for 2 weeks. FBG of the diabetic mice was determined on the day 7 and 14 as described before. On the day 14, the mice were sacrificed under diethyl ether anesthesia after the FBG determination. Blood was collected from the ophthalmic vein and allowed to clot at room temperature for 30 min. The serum was separate by centrifuging at 4000 rpm for 10 min with a refrigerated centrifuge. Liver and kidney were excised. Samples were stored at −20°C until used.

### Assay of GSP

GSP level was measured according to the nitroblue tetrazolium (NBT) colorimetric method using commercial kits purchased from Nanjing Jiancheng Bioengineering Institute (Nanjing, China) [25]. This method based on the ability of the ketoamine group of glycated proteins to reduce tetrazolium salt under alkaline conditions. Fructosamine was used as standard.

### Antioxidant Enzyme Activities

10% solution of tissue homogenate was prepared as follows. Pieces of liver or kidney were homogenized in 9 volumes (w/v) ice-cold saline and centrifuged at 4000 rpm for 8 min. The supernatants were separated and stored at 4°C for analysis. Protein content of tissue homogenate was determined according to the Bradford method [26]. Bovine serum albumin was used as standard. The superoxide dismutase (SOD), catalase (CAT) and glutathione peroxidase (GSH-Px) activities in liver and kidney tissue, the SOD and CAT activities in serum were determined using the respective commercial kits obtained from Nanjing Jiancheng Bioengineering Institute (Nanjing, China). SOD activity was determined by the nitrite method [27]. SOD activity was expressed as units/mg protein or units/mL serum. The activity of CAT was measured using colorimetric method based on the decomposition of hydrogen peroxide (H_2_O_2_) by CAT. The enzyme catalysis was stopped by adding the solution of ammonium molybdate which can react with the rest H_2_O_2_ and give a yellow product. The product can be measured at 405 nm by its absorbance. The CAT activities of tissue or serum were presented as units/mg protein or units/mL serum respectively. GSH-Px activity was also measured according to a colorimetric method developed by Sedlak [28]. Glutathione (GSH) had the ability to decompose H_2_O_2_ using GSH-Px as catalyst. The remaining GSH reacted with dithiobisnitrobenzoic acid to give 2-nitro-5-thidbenzoic acid, which had an absorbance peak at 412 nm. Consumption of GSH was used to calculate the GSH-Px activity. One unit (U) of GSH-Px was defined as the amount that reduces the level of GSH by 1 *μ*mol.

### Extraction, Isolation, and Purification Procedures of Bioactive Constituents from Mulberry Fruit

The ethyl acetate-soluble fraction (MFE) (78.5 g) prepared from dried mulberry fruit (14.7 kg) as described previously was further purified by silica gel chromatography (200–300 mesh, 1150×80 mm) and eluted with a CHCl_3_/MeOH gradient elution (the ratios of CHCl_3_/MeOH were from 100:0 to 0:100). The CHCl_3_/MeOH (100:3) elution (2.25 g) was subjected to a silica gel column (300×30 mm) using hexane/ethyl acetate (100:0 to 0:100) gradient elution. Then the hexane/ethyl acetate (10:1, 350 mg) subfraction was further purified on a semi-preparative HPLC using the Cosmosil 5C18-MS- II, 10ID×250 mm column, which eluted isocratically with 45% methanol in water at a flow rate of 3.0 mL/min. Compound **23** (9.0 mg) was obtained at a retention time of 19.6 min. The hexane/ethyl acetate (10:2) elution (340 mg) was applied to a Sephadex LH-20 column (700 ×10 mm) eluted with MeOH/H_2_O (80:20), compounds **14** (16.0 mg) and **12** (40.0 mg) were purified. The CHCl_3_/MeOH (100:4, 6.89 g) eluent of the ethyl acetate fraction was further chromatographed on a silica gel column (500×40 mm) using hexane/ethyl acetate (100:0 to 0:100) gradient elution, Compound **21** (40.6 mg) was obtained from hexane/ethyl acetate(9:1) elution. Then the subfraction hexane/ethyl acetate (10:1, 100 mg/kg mg) was further isolated using a Sephadex LH-20 column (700×10 mm) eluted with CHCl_3_/MeOH (2:1). Compounds **22** (16.5 mg) and **25** (15.8 mg) were obtained. The subfraction hexane/ethyl acetate (8:1, 385.0 mg) was subjected to a Sephadex LH-20 column (700 ×10 mm) using CHCl_3_/MeOH (2:1) as eluting solvent, compounds **17** (91.6 mg/kg mg) and **19** (19.6 mg) were purified. The subfraction hexane/ethyl acetate (5:1, 349.0 mg) was further isolated with ODS column (300×20 mm) eluted with MeOH/H_2_O gradient elution (the ratios of MeOH/H_2_O were from 10:100 to 100:0). Compounds **24** (18.0 mg) and **13** (8.0 mg) were obtained from the MeOH/H_2_O (40:60) eluent and MeOH/H_2_O (50:50) eluent, respectively. The CHCl_3_/MeOH (100:5, 11.52 g) eluent of the ethyl acetate fraction was subjected to a silica gel column (600×40 mm) using CHCl_3_/ CH_3_COCH_3_ (100:0 to 0:100) gradient elution. Cubic crystals of compound **18** (850 mg) were obtained from the CHCl_3_/CH_3_COCH_3_ (20:1) elution. The CHCl_3_/ CH_3_COCH_3_ (50:1, 59.3 mg) elution was further purified with a semi-preparative HPLC column (Cosmosil 5C18-MS- II, 10ID×250 mm) using 30% methanol in water (containing 0.1% CF_3_COOH) as mobile phase at a flow rate of 3.0 mL/min. Compound **20** (10.7 mg) was purified at a retention time of 25.1 min. The CHCl_3_/MeOH (100:10, 3.58 g) eluent of the ethyl acetate fraction was applied to a Sephadex LH-20 column (800×20 mm) eluted with CHCl_3_/MeOH (2:1 to 1:1). Compound **1** (150 mg) was obtained from the CHCl_3_/MeOH (1:1) elution. The CHCl_3_/MeOH (2:1) elution was further purified with a semi-preparative HPLC column (Cosmosil 5C18-MS- II, 10ID×250 mm) which eluted isocratically with 40% methanol in water at a flow rate of 3.0 mL/min. Compounds **9** (10.8 mg), **10** (22.0 mg/kg mg), **3** (15.2 mg/kg mg) and **7** (54.0 mg/kg mg) were obtained at retention times of 14.5, 19.6, 22.3 and 37.9 mg/kg mg/kg min, respectively. The CHCl_3_/MeOH (5:1, 4.18 g) eluent of the ethyl acetate fraction was chromatographed on an ODS column (300×30 mm) eluted gradiently with MeOH/H_2_O (the ratios of MeOH/H_2_O were from 10:100 to 100:0). The MeOH/H_2_O (30:70, 2.5 g) elution was further isolated over a Sephadex LH-20 column (800×20 mm) eluted with MeOH/H_2_O (70:30) to yield compounds **4** (1.61 g) and **2** (202.8 mg). Then the MeOH/H_2_O (40:60, 212.3 mg) elution was also subjected to a Sephadex LH-20 column (700×10 mm) eluted with MeOH/H_2_O (80:20) and compound **8** (18.2 mg/kg mg) was obtained. The CHCl_3_/MeOH (2:1, 1.92 g) eluent of the ethyl acetate fraction was further applied to an ODS column (300×20 mm) eluted with MeOH/H_2_O (the ratios of MeOH/H_2_O were from 10:100 to 100:0). The MeOH/H_2_O (50:50, 273.8 mg) elution was subjected to a semi-preparative HPLC column which was eluted isocratically with 40% methanol in water (containing 0.1% CF_3_COOH) at a flow rate of 3.0 mL/min. Compounds **15** (31.0 mg) and **16** (14.2 mg) were obtained at retention times of 11.6 and 27.1 min respectively. The CHCl_3_/MeOH (1:1, 1.82 mg/kg mg/kg mg/kg g) eluent of the ethyl acetate fraction was chromatographed over an ODS column (300×20 mm) eluted with MeOH/H_2_O (from 10:100 to 100:0). The MeOH/H_2_O (20:80, 150 mg) elution was subjected to a Sephadex LH-20 column (700×10 mm) eluted with MeOH/H_2_O (60:40), to yield compound **5** (6.0 mg). Then the MeOH/H_2_O (30:70, 350 mg) elution was also chromatographed using a Sephadex LH-20 column (700×10 mm) which eluted with MeOH/H_2_O (60:40) to afford compounds **6** (8.0 mg/kg mg) and **11** (8.2 mg ).

All of the pure compounds isolated from mulberry fruit were evaluated for their antioxidant activity using the DPPH radical-scavenging and superoxide anion radical-scavenging assay, and inhibitory activity against α-glucosidase.

### High Performance Liquid Chromatography-Photodiode Array Detector (PDA) Analysis

The separation of MFE was performed on the Sepax Amethyst C18-H analytical column (4.6×250 mm, 5 *μ*m) using the Waters chromatographic system at a flow rate of 1.0 mL/min. The mobile phase composed of phase A (methanol: water: acetic acid  = 5: 95: 0.1) and phase B (methanol: acetic acid  = 100: 0.1). The linear gradient (0–53 min) was performed using the timetable as follows: phase A was held constant for 5 mg/kg mg/kg min, then phase B increased to 50% in 25 min, to 70% in 10 min, to 80% in 5 min, to 100% in another 5 min followed by A increased to 100% in 3 min and column reconditioning for 10 more minutes for the next injection. The column oven was set at 40°C. The UV spectra were recorded in the 210–400 nm range. Compounds purified from MFE were used as standard reference. The peaks were identified by comparison of their retention times and extracted UV spectrum from PDA with those of the reference standards.

### Statistical Analysis

Experimental data were expressed as mean ± standard deviation (SD). The statistical analysis was performed using the SPSS software (Version 20 for windows, IBM, Chicago, IL). Differences between groups were analyzed by one-way analysis of variation (ANOVA), followed by Dunett's test and least significant difference (LSD) test. Difference with p value <0.05 was considered to be significant.

## Results and Discussion

### Antioxidant, α-Glucosidase Inhibitory Activities and Total Phenolic, Total Flavonoids Contents of the Extracts from Mulberry Fruit

The EtOAc-soluble extract (MFE) was found to be the most potent fraction among the 70% ethanol extract, hexane, CHCl_3_, EtOAc, n-BuOH and H_2_O-soluble extract fractions in the *in vitro* antioxidant and α-glucosidase inhibitory assays, which were shown in **Table 1**. Chemical analysis showed that MFE prepared from mulberry fruit was rich in phenolics and flavonoids (**Table 2**). It could be supposed that the main bioactive components which may stand for its antioxidant and antidiabetic activities of the 70% ethanol extract from mulberry fruit were basically in the EtOAc-soluble extract (MFE). Thus MFE was selected to investigate the antioxidant and hypoglycemia effect *in vivo*. The further study on chemical composition was also performed.

**Table 1:** DPPH Radical-scavenging, Superoxide anion Radical-scavenging Activities and α-glucosidase Inhibitory Activity of the Extracts from Mulberry Fruit.a, b

	EC_50_ (mg/L)		IC_50_ (mg/L)
Sample	DPPH	Superoxide anion	α-Glucosidase
70% EtOH extract	335.56±5.13	921.83±55.82	367.74±24.35
Hexane-soluble	575.89±18.08	327.43±21.48	NA
CHCl_3_-soluble	375.90±9.48	323.92±15.11	102.24±5.81
EtOAc-soluble	71.12±1.72	82.37±4.6	72.01±4.18
n-BuOH-soluble	206.33±5.68	389.47±12.85	447.35±9.87
Water-soluble	623.86±32.4	269.1±9.27	275.68±24.13
Acarbose	NA	NA	77.05±6.10
Gallic acid	4.95±0.24	5.21±0.27	NA

a Values are the mean ± SD; n = 3. ^b^ DPPH used in the DPPH radical-scavenging assay of the extracts was 2,2-Di(4-tert-octylphenyl)-1-picrylhydrazyl (Sigma-Aldrich). NA, no assay.

**Table 2:** Total Phenolic Content (TPC) and Total Flavonoid Content (TFC) of the Extracts from Mulberry Fruit.^a^

Sample	TPC (mg GAE/g)	TFC (mg RE/g)
70% EtOH extract	32.24±0.35	18.08±0.87
Hexane-soluble	17.88±1.05	NA
CHCl_3_-soluble	41.85±1.10	43.09±2.29
EtOAc-soluble	194.67±2.88	261.78±6.26
n-BuOH-soluble	71.26±2.12	60.89±2.22
Water-soluble	24.87±0.22	9.68±1.51

a Values are the mean ± SD; n = 3. GAE, gallic acid equivalent. RE, rutin equivalent; NA, no assay.

### Effect of the MFE on the FBG and GSP of STZ-induced Diabetic Mice

The average FBG level of the MFE (200 mg/kg) group was significantly lower than that of the control group (p<0.05) on day 14, as shown in **Table 3**. For the positive control group administrated Metformin (300 mg/kg), the average FBG level of the mice was significantly lower than that of the control group (p<0.01) on day 7 and day 14. The GSP levels in the MFE (200 mg/kg) and Metformin (300 mg/kg) groups were 6.44±0.78 and 6.06±0.47 mmol/L, respectively. They were significantly lower than that of the control group (7.25±0.33 mmol/L, p<0.05). GSP is increased in diabetes mellitus owing to the persistently high level of blood glucose, and it reflects the degree of blood glucose during a period of 2–3 weeks [25]. These results showed that MFE decreased the level of blood glucose in STZ induced diabetic mice.

**Table 3:** Effect of the MFE on the Fasting Blood Glucose (FBG) and Glucosylated Serum Protein (GSP) of STZ-induced Diabetic Mice.^a^

	FBG (mmol/L)			GSP (mmol/L)
Group	day 0	day 7	day 14	day 14
Control	24.36±7.72	26.13±6.92	27.27±6.26	7.25±0.33
MFE (100mg/kg)	24.91±7.57	22.74±7.82	25.08±5.76	6.94±0.69
MFE (200mg/kg)	24.08±7.65	20.51±6.98	19.66±7.40b	6.44±0.78b
Metformin (300mg/kg)	25.66±5.95	15.44±4.57c	17.38±5.31c	6.06±0.47b

a Values are the mean ± SD; n = 8. ^b^ p<0.05 versus the control group. ^c^ p<0.01 versus the control group.

### Effect of the MFE on Antioxidant Enzymes Activities of STZ-induced Diabetic Mice

**Table 4** shows the SOD, CAT activities in serum and the SOD, CAT, GSH-Px activities in kidney and liver. Compared to the diabetic control group, the SOD activities of the MFE (200 mg/kg) and Metformin (300 mg/kg) groups increased significantly in serum (p<0.05). MFE (100, 200 mg/kg) and Metformin (300 mg/kg) groups produced significant increase of the CAT activity in serum (p<0.01). Significant increase of the SOD and CAT activities in kidney was observed in the MFE (200 mg/kg) and Metformin (300 mg/kg) groups (p<0.05), compared to the diabetic control group. The GSH-Px activity of kidney in MFE (200 mg/kg) group increased significantly compared to the diabetic control group. In the case of the Metformin (300 mg/kg) group, the GSH-Px activity increased compared to the diabetic control group, but there was no significant difference (p>0.05).

**Table 4:** Effect of the MFE on Antioxidant Enzyme Activities of STZ-induced Diabetic Mice.^a^

	Diabetic	MFE		Metformin
Para meters	Control	100 mg/kg	200 mg/kg	300 mg/kg
Serum				
SOD	211.90±23.88	228.23±33.20	237.26±18.06b	251.29±28.78b
CAT	18.00±4.73	42.48±13.11c	56.60±16.18c	50.53±17.87c
Kidney				
SOD	219.19±23.18	239.74±16.97	254.69±30.78b	246.56±24.80b
CAT	16.41±3.52	18.24±2.76	23.33±5.58b	21.85±3.90b
GSH-Px	219.04±24.96	233.26±11.43	252.26±32.60b	240.36±40.31
Liver				
SOD	149.88±9.31	150.72±15.80	166.61±10.20c	145.03±13.79
CAT	25.53±2.91	30.02±5.12b	34.73±6.71c	27.07±2.74
GSH-Px	275.20±17.09	296.11±37.63b	308.74±37.42b	275.78±21.02

a Values are the mean ± SD; n = 8. ^b^ p<0.05 versus the control group. ^c^ p<0.01 versus the control group.

The levels of SOD, CAT and GSH-Px of liver increased significantly in MFE (200 mg/kg) group (p<0.05 or p<0.01) compared to the diabetic control group. Significant increase of CAT and GSH-Px levels in liver was also observed in the MFE (100 mg/kg) group (p<0.05). No significant difference in SOD, CAT and GSH-Px activities of liver was observed between the Metformin (300 mg/kg) and diabetic control groups (p>0.05).

The results of the present study indicated that MFE increased SOD, CAT, GSH-Px activities and consequently increased the antioxidant activities of organs and serum. Yang *et al* [10] reported that feeding freeze-dried powder of mulberry fruit (*M. alba* L.) improved the level of antioxidant capacity through significantly increased SOD and GSH-Px activity in the liver and blood of hyperlipidemic rats. Hong *et al* [29] reported that mulberry fruit strengthened the antioxidant defense systems through increased activity of antioxidant enzymes, such as CAT and GSH-Px in the erythrocytes of the STZ-induced diabetic mice. Therefore, these previous studies and the results of the present study suggest that these potential and beneficial effects of mulberry fruit extract on the antioxidant enzymes activities of STZ-induced diabetic mice might be attributed to its polyphenol enhanced extract (MFE). Furthermore, the beneficial effects of MFE seem to be due to the rich content of phenolics including flavonoids.

### Structure Identification of the Purified Compounds

**Figure 1:**
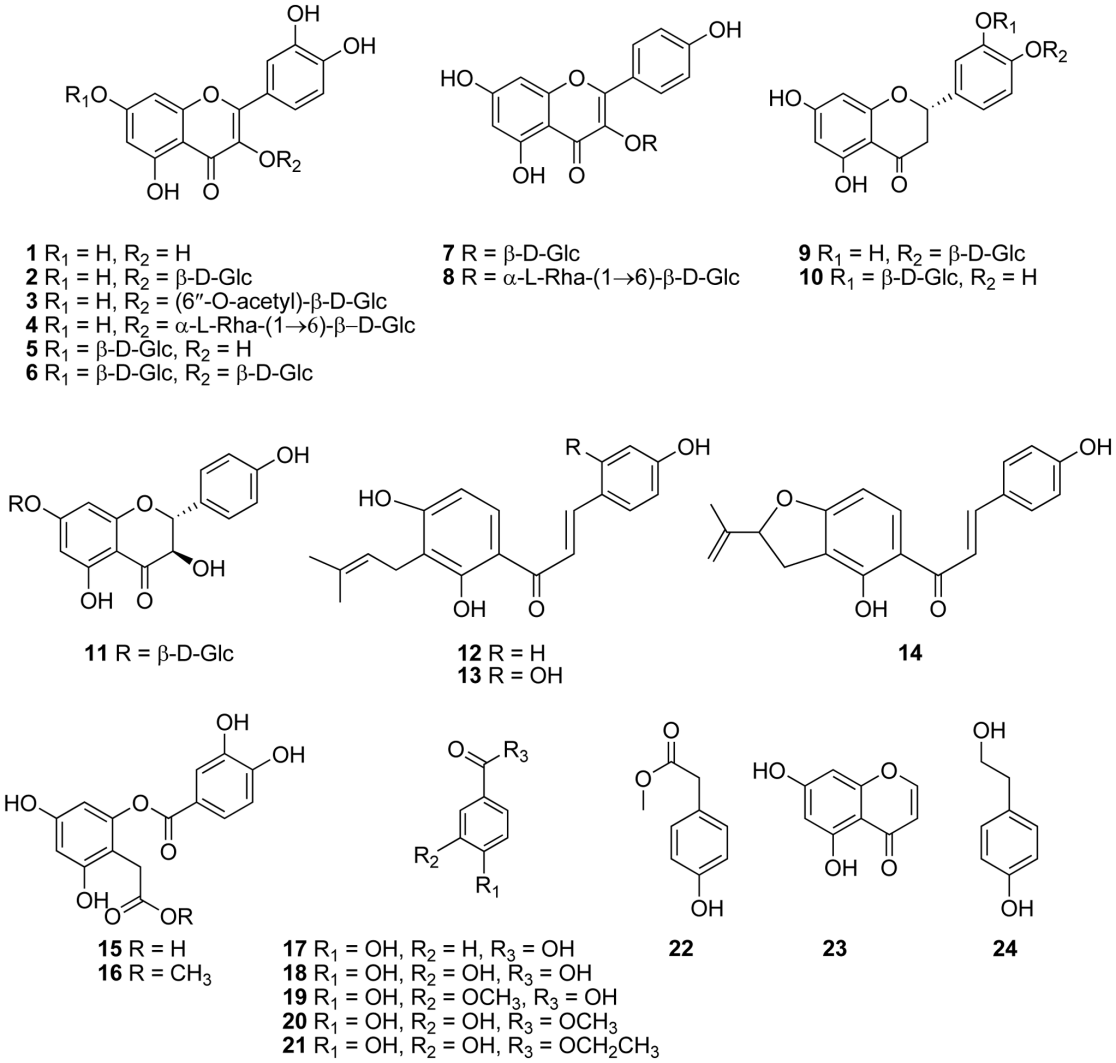
Chemical structure of compounds 1−24 isolated from mulberry fruit (*Morus alba* L.)

Further detailed fractionation of MFE led to the isolation of 25 phenolic compounds (**1−25**). The chemical structures of compounds **1−24** were shown in **Figure 1**.

Compounds **1**−**8** were flavonoids with different sugar moieties or substitution patterns. Compound **1** was obtained as yellow power. The molecular formula of **1** was inferred as C_15_H_10_O_7_ on the basis of analysis of ESI-MS and ^1^H and ^13^C NMR spectra. In the ^1^H NMR spectrum, an ABX spin-coupling system was ascribed to the three protons of the B-ring of a flavone, with signals at 7.67 (H, d, *J* = 2.0 Hz), 7.54 (H, dd, *J* = 8.5, 2.0 Hz), and 6.88 ppm (H, d, *J* = 8.5 Hz), respectively. The signals at 6.41 (H, d, *J* = 1.6 Hz) and 6.19 ppm (H, d, *J* = 1.6 Hz) were meta-substituted protons of the A-ring. The signal at 12.50 ppm (H, s) was the characteristic signal of the hydroxyl at C-5 of a flavone. Therefore, **1** was identified as quercetin. Compound **2** was obtained as yellow powder. The ESI-MS spectrum showed the ion [M + H]^+^ at 465, and the molecular formula of C_21_H_20_O_12_ was inferred from ^1^H and ^13^C NMR. Compared with the NMR data of **1**, the aglycone of **2** was quercetin. The signal at 5.46 ppm (H, d, *J* = 6.9 Hz) in the ^1^H NMR was an anomeric proton showed that there was a sugar unit in the molecule. There were 21 carbon signals in the ^13^C NMR. From the signals in ^13^C and ^1^H NMR, the sugar was identified as a glucose unit. The β-configuration of the glucose was drawn from the coupling constant of the anomeric proton. At the basis of the above analysis, **2** was identified as quercetin-3-O-β-D-glucopyranoside, which was identical to the published data in the literature [30]. Compound **3** had the same aglycone and sugar moiety compared to compound **2**. The molecular formula C_23_H_22_O_13_ can be inferred from its ESI-MS, ^1^H and ^13^C NMR. The proton signal at 1.72 ppm (3H, s) in the ^1^H NMR spectrum, carbon signals at 169.9 and 20.1 ppm in the ^13^C NMR spectrum suggested the presence of one acetyl group in the molecule. The acetyl group was linked at C-6′′ according to its ^1^H, ^13^C and literature data. Therefore, **3** was identified as quercetin 3-O-(6′′-O-acetyl)-β-D-glucopyranoside [31]. Compound **4** was identified as quercetin 3-O-β-D-rutinoside with a rutinose linked to C-3 of **1** (quercetin). The ^1^H and ^13^C NMR spectrum of compound **5** were similar to those of **2** and also had aglycone as quercetin and a glucose unit in its molecule. The difference between them was the glucose unit of **5** linked to C-7 instead of C-3. By comparison with the literature [32], **5** was identified as quercetin 7-O-β-D-glucopyranoside. Compound **6** had two glucose units linked to C-3 and C-7 respectively was identified as quercetin 3,7-di-O-β-D-glucopyranoside [33]. Compounds **7** and **8** had the same aglycone in their structures. The AA′BB′ spin-coupling system with the signals at 8.03 (2H, d, *J* = 8.7 Hz) and 6.89 ppm (2H, d, *J* = 8.7 Hz) in their ^1^H NMR spectrums was ascribed to the four protons of the B-ring. The signals at 6.41 (H, d, *J* = 1.5 Hz) and 6.20 ppm (H, d, *J* = 1.5 Hz) were meta-substituted protons of the A-ring. The characteristic signal of the hydroxyl at C-5 of flavone was observed in both **7** and **8**. In their ^13^C NMR spectra, two resonances at 130.9 and 115.1 ppm representing four aromatic carbons showed that there was a 1,4-disubstituted benzene fragment in the molecule. Therefore, the aglycone of **7** and **8** was identified as kaempferol. The different sugar moieties of **7** and **8** were further identified as glucose and rutinose respectively according to their ^1^H and ^13^C NMR. On the basis of the above analysis, they were identified as kaempferol 3-O-β-D-glucopyranoside (**7**) [34] and kaempferol 3-O-β-D-rutinoside (**8**) [35] respectively.

Compounds **12−14** were derivatives of chalcones. The ESI-MS of **12** showed the ion [M + Na]^+^ at 347 suggesting the molecular formula of C_20_H_20_O_4_, which confirmed by ^1^H, ^13^C NMR and DEPT spectra. In the high field of the ^1^H NMR spectrum, proton signals at 1.62 (3H, s), 1.72 (3H, s), 3.23 (2H, m) and 5.17 ppm (1H, t-like m) were ascribed to a prenyl group. In the down field, an AA′BB′ spin-coupling system with signals at 7.75 (2H, d, *J* = 8.7 Hz) and 6.84 ppm (2H, d, *J* = 8.7 Hz) was assigned to the B-ring. Ortho-substituted protons with signals at 8.04 (H, d, *J* = 8.9 Hz) and 6.47 ppm (H, d, *J* = 8.9 Hz) were ascribed to the A-ring. The singlet at 14.01 ppm was one chelated hydroxyl group at C-2′ of chalcone. The signals in the ^13^C NMR spectra showed 20 carbons in the molecule. One carbonyl signal appeared at 191.8 ppm, and two olefinic carbons appeared at 144.1 and 117.7 ppm. On the basis of the analysis of the above data, **12** was identified as isobavachalcone, which was identical to the reported data [36]. Compound **13** was also obtained as amorphous yellow powder. The ESI-MS gave the ion [M + H]^+^ at 341 corresponding to the molecular formula of C_20_H_20_O_5_. When compared to **12**, an ABX instead of AA′BB′ spin-coupling system appeared in the ^1^H NMR spectrum with signals at 7.71 (H, d, *J* = 8.9 Hz), 6.39 (H, br. s), and 6.32 ppm (H, dd, *J* = 8.6, 1.2 Hz), and was assigned to the B-ring. The different substitution pattern of the B-ring was supported by ^13^C NMR spectrum. On the basis of the above analysis, **13** was identified as 2,4,2′,4′,-tetrahydroxy-3′-(3-methyl-2-butenyl)-chalcone (morachalcone) [37]. The ^1^H NMR spectra of compound **14** showed a vinyl methyl proton at 1.83 ppm (3H, br. s), two exocyclic methylene protons at 4.94 (H, br. s) and 4.75 ppm (H, br. s), a set of methylene protons at 3.11 (H, dd, *J* = 14.2, 3.2 Hz) and 2.90 ppm (H, dd, *J* = 14.2, 8.1 Hz), a methine proton at 4.40 (H, dd, *J* = 8.0, 3.1 Hz) due to an ether-ring side chain. In the ^13^C NMR spectra, a carbon bearing a hydroxyl group appeared at 76.8 ppm. Thus, **14** was identified as (2E)-1-[2,3-dihydro-4-hydroxy-2-(1-methylethenyl)-5-benzofuranyl]-3-(4-hydroxyphenyl)-1-propanone, as reported previously [38].

Compounds **9−11** were derivatives of flavanones or flavanonols. The molecular formula of them was inferred as C_21_H_22_O_11_ from their ESI-MS and ^13^C NMR. Analysis of their ^1^H and ^13^C NMR revealed that the aglycone was eriodictyol, and there was a sugar unit in their molecular. The minor difference between their ^13^C NMR suggested the different substituted position of the sugar unit. By comparing their ESI-MS and ^1^H and ^13^C NMR with the published literatures, **9** and **10** were identified as 5,7,3′-trihydroxy-flavanone-4′-O-β-D-glucopyranoside [39] and 5,7,4′-trihydroxy-flavanone-3′-O-β-D-glucopyranoside [40] respectively. **11** was identified as dihydrokaempferol 7-O-β-D-glucopyranoside [41] according to its ESI-MS and ^1^H and ^13^C NMR spectrum.

Compounds **15−22** were derivatives of phenolic acid, which were identified as 2-O-(3,4-dihydroxybenzoyl)-2,4,6-trihydroxyphenylacetic acid (**15**) [42], 2-O-(3,4-dihydroxybenzoyl)-2,4,6-trihydroxyphenylmethylacetate (jaboticabin) (**16**) [42], p-hydroxybenzoic acid (**17**), protocatechuic acid (**18**), 3-methoxy-4-hydroxybenzoic acid (vanillic acid) (**19**), protocatechuic acid methyl ester (**20**), protocatechuic acid ethyl ester (**21**), 4-hydroxyphenylacetic acid methyl ester (**22**), respectively, according to their ESI-MS and ^1^H and ^13^C NMR data.

Compounds **23**−**25** were identified as 5,7-dihydroxychromone (**23**), 2-(4-hydroxyphenyl)ethanol (tyrosol) (**24**), pyrocatechol (**25**), respectively, on the basis of MS and NMR data.

All the 25 compounds were isolated from mulberry fruit for the first time.

#### HPLC-PDA Analysis

**Figure 2:**
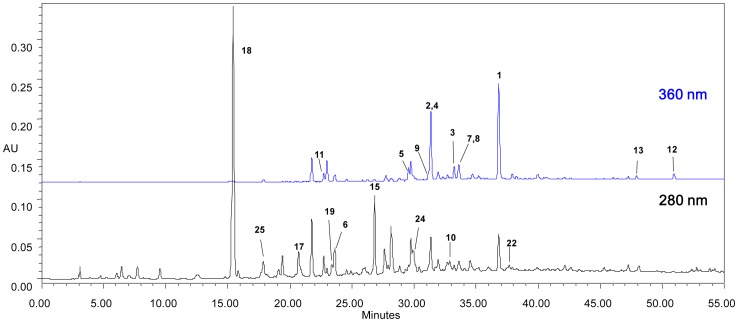
HPLC-PDA chromatogram of mulberry fruit (*Morus alba* L.) polyphenol enhanced extract.

In the HPLC-PDA analysis of MFE, 20 of the 25 isolated compounds were ascribed using the authentic samples as shown in **Figure 2** **.** The ascribed compounds in the phenolic HPLC fingerprints could be considered as characteristic profile of mulberry fruit. Besides, compounds **1**, **2**, **4**, **15**, **17**, **18** were found to be the major constituents isolated taking into consideration of both the peak areas and the yield.

### Antioxidant Activities

*In vitro* DPPH radical-scavenging and superoxide anion radical-scavenging activities of the isolated compounds are summarized in **Table 5**. In the DPPH radical-scavenging assay, **1** (quercetin) and its derivatives (**2**−**6**) show significant radical-scavenging activity with lower EC_50_ values (7.89 − 11.89 *μ*M ) than ascorbic acid (EC_50_  =  33.46±1.20 *μ*M). **7** and **8** were glycosides of kaempferol, showed lower radical-scavenging activity with EC_50_ values of 152.47±5.90 and 301.24±13.84 *μ*M, respectively, compared to the quercetin derivatives. Among the flavanones and flavanonols derivatives (**9−11**), **11** showed the highest radical-scavenging ability with an EC_50_ value of 6.46±0.39 *μ*M, **9** and **10** showed lower radical-scavenging ability with EC_50_ values of 79.38±4.74 and 221.61±13.79 *μ*M, respectively. All of the chalcones (**12−14**) displayed low DPPH radical-scavenging activity with EC_50_ >500 *μ*M. On the basis of analysis of the structure-activity relationship of these flavonoids in the DPPH radical-scavenging assay, it may be suggested that the presence of ortho-dihydroxy (catechol) or 3-OH substitutions in the molecule impart their high DPPH radical-scavenging ability. This can be illustrated by, for example, the relative lower EC_50_ values of compounds **1−6**, **11** and the relative higher EC_50_ values of compounds **7**−**10**. This observation is well supported by previous reports [43].

**Table 5:** DPPH Radical-scavenging and Superoxide anion Radical-scavenging Activities of The Isolated Compounds 1–25.^a^

	EC50 (*μ*M)	
Compound b	DPPH	Superoxide anion
**1**	11.89±0.72	27.94±0.59
**2**	7.89±0.50	38.24±0.92
**3**	11.75±1.04	45.09±1.66
**4**	9.76±0.61	30.62±0.99
**5**	9.80±0.53	24.62±1.67
**6**	9.18±0.97	50.69±6.70
**7**	301.24±13.84	121.91±5.49
**8**	152.47±5.90	196.45±7.98
**9**	79.38±4.74	90.70±8.28
**10**	221.61±13.79	194.24±8.12
**11**	6.46±0.39	58.33±2.39
**12**	>500	149.56±9.10
**13**	>500	191.19±15.0
**14**	>500	174.63±9.72
**15**	7.87±0.73	15.17±0.70
**16**	10.76±0.94	37.49±2.62
**18**	11.88±0.69	100.72±8.27
**20**	10.69±0.34	214.35±16.91
**21**	9.04±0.21	605.75±25.81
**24**	>500	417.21±23.90
**25**	6.74±0.28	45.79±2.97
Gallic acid	5.18±0.34	10.12±0.39
Ascorbic acid	33.46±1.20	>500

a Values are the mean ± SD; n = 3.

b Compounds **17**, **19**, **22** and **23** did not show DPPH radical-scavenging or superoxide anion radical-scavenging activity (EC50 >500 *μ*M).

The DPPH radical-scavenging activity order of protocatechuic acid, the major constituents of mulberry fruit, and its derivatives was shown to be **21**>**20**>**18**>**17** or **19**, suggesting that the presence of a catechol group in the compounds **18**, **20**, **21** enhances their ability to scavenge DPPH radical, and the introduction of alkyl groups in the carboxylic acid led to the increase of antioxidant activity in DPPH radical-scavenging assay. This observation is well supported by previous studies [44], [45]._ENREF_34 The two depsides, compounds **15** and **16**, which are rarely found in higher plants, had low EC_50_ values of 7.87±0.73 and 10.76±0.94 *μ*M. These results were consistent with earlier studies [46]. As expected, compound **25** showed strong ability to scavenge DPPH radical with a low EC_50_ value (6.74±0.28 *μ*M). Compounds **22**−**24** displayed weak DPPH radical-scavenging activity even at a high concentration tested.

The superoxide anion radical-scavenging assay showed that compound **2** had the highest radical-scavenging activity with an EC_50_ value of 15.17±0.70 *μ*M, followed by **5** (EC_50_  = 24.62±1.67 *μ*M) and **1** (EC_50_  = 27.94±0.59 *μ*M). On the basis of the superoxide anion radical-scavenging assay results obtained, some observations can be made. First, in the case of protocatechuic derivatives, the activity order was shown as **18**>**20**>**21**, in contrast to the results of DPPH assay and previous study [45], indicated that the introduction of alkyl groups in the carboxylic acid produced negatively influence on the antioxidant activity in superoxide anion radical-scavenging assay. The different results obtained from these two assays may be explained by the different mechanisms of these assays, and suggested that combined assay methods should be adopted in the screening and evaluation of bioactivity components from nature materials. Second, the presence of ortho-dihydroxy (catechol) or 3-OH (in flavonoids) substitutions enhances their superoxide anion radical-scavenging activity, which was the same to DPPH radical-scavenging assay.

### α-Glucosidase Inhibitory Activity

All compounds isolated were investigated for inhibitory activity against α-glucosidase. The inhibitory profiles of compounds **1−25** were summarized in **Table 6**.

**Table 6:** α-Glucosidase Inhibitory Activities of the Isolated Compounds 1−25.^a^

Compound b	IC50 (*μ*M)
**1**	8.57±0.57
**2**	165.35±11.55
**3**	310.10±12.73
**4**	165.64±11.73
**5**	369.89±31.24
**6**	600.31±45.88
**7**	197.05±9.26
**8**	178.34±0.35
**9**	408.91±38.70
**10**	727.97±67.74
**11**	932.51±98.75
**12**	67.30±5.51
**13**	49.96±3.18
**14**	225.29±4.79
**15**	116.14±6.75
**16**	31.70±3.38
Acarbose	119.15±4.90

a Values are the mean ± SD; n = 3. ^b^ Compounds **17**−**25** did not show α-glucosidase inhibitory activity (IC50 >1000 *μ*M).

All of the 14 flavonoids exhibited potent inhibitory activity against α-glucosidase. Among the flavonoids, **1** (quercetin) (IC_50_  = 8.57±0.57 *μ*M) was the most active one, followed by **13** (IC_50_  = 49.96±3.18 *μ*M) and **12** (IC_50_  = 67.30±5.51 *μ*M). The activity of these flavonoids was significantly affected by subtle changes of the structure. On the basis of the analysis of these changes, two conclusions can be draw from the results obtained. First, glycosylation of the hydroxyl(s) on the 3- or/and 7-position significantly decreased the inhibitory activity. This could be elucidated by the following. Compound **1** had much lower IC_50_ value (8.57±0.57 *μ*M) than **4** (165.64±11.73 *μ*M), **2** (165.35±11.55 *μ*M), **3** (310.10±12.73 *μ*M), **5** (369.89±31.24 *μ*M) and **6** (600.31±45.88 *μ*M). Glycosylation at both 3- and 7-OH of **6** led to the lowest inhibition. Besides, a great difference in inhibition between **5** (369.89±31.24 *μ*M) and **2** (165.35±11.55 *μ*M) seemed to indicate that 7-OH is more important to the inhibitory. Second, increase in the number of the hydroxyl group on the B-ring enhances the inhibitory activity of flavonoid. Compared to **7** (197.05±9.26 *μ*M) and **8** (178.34±0.35 *μ*M), compounds **2** (165.35±11.55 *μ*M) and **4** (165.64±11.73 *μ*M) showed higher inhibitory activity. The above-mentioned conclusions were well supported by previous studies [47], [48] showing that the 3-OH, the hydroxyl substitution on the B-ring enhanced the inhibitory activity. In these literatures, 5-OH also enhanced the inhibitory activity. In present study, we found that glycosylation of 7-OH was unfavorable to the inhibitory activity.

In the case of chalcones, **12** and **13** showed significant inhibitory activities higher than that of clinically used acarbose (IC_50_  = 119.15±4.90 *μ*M), with IC_50_ values of 67.30±5.51 and 49.96±3.18 *μ*M, respectively. Previous literatures reported that the prenylated chalcone with a catechol moiety in the B-ring and a resorcinol moiety in the A-ring was the most effective chalcone derivative [49]. Significantly decrease in the inhibitory activity of **14** may be attributed to lose of 4′-OH resulted from the cyclization of the hydroxyl group onto the pendant allyl group.

Both of the two depsides, **15** and **16** showed higher inhibitory activity than acarbose. The inhibitory ability sequence against α-glucosidase was **16** (IC_50_  = 31.70±3.38 *μ*M) >**15** (IC_50_  = 116.14±6.75 *μ*M) > acarbose (IC_50_  = 119.15±4.90 *μ*M). Comparing the inhibitory activity of **15** and **16**, we found that the inhibitory activity increased considerably with the introduction of methyl group in the carboxylic acid. This result may indicate that increase of lipophilicity of depside enhanced the inhibitory activities. Similar phenomena were observed in the investigation of flavonoids inhibition against α-glucosidase, in literature study previously [50]. **15** and **16** represent a novel class of depsides that has been reported recently [42], [46], [51]–[53]. Bioactivity studies *in vitro* revealed that they had antioxidant activity [46], [51], anticancer activity [46], [51], and antibacterial activity [54]. In the present research, **15** and **16** were found to have significant inhibitory activity against α-glucosidase. To the best of our knowledge, this is the first ever report on the α-glucosidase inhibition of depsides. The high potency of these pure compounds for radical-scavenging and α-glucosidase inhibitory activities *in vitro* may stand for, at least in part, the high antioxidant capacity and hypoglycemic activity, respectively, of mulberry fruit extract. The mechanisms of action of compounds in diabetic prevention are worthy of further research. The above results will promote the usage of mulberry fruit as herb or functional food.

## References

[1] Sato Y, Hotta N, Sakamoto N, Matsuoka S, Ohishi N, et al (1979) Lipid peroxide level in plasma of diabetic patients. Biochem Med 21: 104–107. 454385 10.1016/0006-2944(79)90061-9

[2] Kang M-H, Lee MS, Choi M-K, Min K-S, Shibamoto T (2012) Hypoglycemic Activity of Gymnema sylvestre Extracts on Oxidative Stress and Antioxidant Status in Diabetic Rats. J Agric Food Chem 60: 2517–2524. 22360666 10.1021/jf205086b

[3] Sharma R, Buras E, Terashima T, Serrano F, Massaad CA, et al (2010) Hyperglycemia Induces Oxidative Stress and Impairs Axonal Transport Rates in Mice. Plos One 5: e13463. 20976160 10.1371/journal.pone.0013463 PMC2956689

[4] Velioglu YS, Mazza G, Gao L, Oomah BD (1998) Antioxidant Activity and Total Phenolics in Selected Fruits, Vegetables, and Grain Products. J Agric Food Chem 46: 4113–4117.

[5] Larson RA (1988) The antioxidants of higher plants. Phytochemistry 27: 969–978.

[6] Kerem Z, Bilkis I, Flaishman MA, Sivan L (2006) Antioxidant Activity and Inhibition of α-Glucosidase by trans-Resveratrol, Piceid, and a Novel trans-Stilbene from the Roots of Israeli Rumex bucephalophorus L. J Agric Food Chem. 54: 1243–1247. 10.1021/jf052436+ 16478243

[7] Mai TT, Thu NN, Tien PG, Van Chuyen N (2007) Alpha-glucosidase inhibitory and antioxidant activities of Vietnamese edible plants and their relationships with polyphenol contents. J Nutr Sci Vitaminol 53: 267–276. 17874833 10.3177/jnsv.53.267

[8] Ranilla LG, Kwon Y-I, Apostolidis E, Shetty K (2010) Phenolic compounds, antioxidant activity and in vitro inhibitory potential against key enzymes relevant for hyperglycemia and hypertension of commonly used medicinal plants, herbs and spices in Latin America. Bioresour Technol 101: 4676–4689. 20185303 10.1016/j.biortech.2010.01.093

[9] Zhang W, Han F, He J, Duan C (2008) HPLC-DAD-ESI-MS/MS analysis and antioxidant activities of nonanthocyanin phenolics in mulberry (Morus alba L.). J Food Sci 73: C512–C518. 19241543 10.1111/j.1750-3841.2008.00854.x

[10] Yang X, Yang L, Zheng H (2010) Hypolipidemic and antioxidant effects of mulberry (Morus alba L.) fruit in hyperlipidaemia rats. Food Chem Toxicol 48: 2374–2379. 20561945 10.1016/j.fct.2010.05.074

[11] Huang HP, Shih YW, Chang YC, Hung CN, Wang CJ (2008) Chemoinhibitory effect of mulberry anthocyanins on melanoma metastasis involved in the Ras/PI3K pathway. J Agric Food Chem 56: 9286–9293. 18767864 10.1021/jf8013102

[12] Jeong Ji C, Jang Sang W, Kim Thae H, Kwon Chae H, Kim Yong K (2010) Mulberry fruit (Moris fructus) extracts induce human glioma cell death in vitro through ROS-dependent mitochondrial pathway and inhibits glioma tumor growth in vivo. Nutr Cancer 62: 402–412. 20358478 10.1080/01635580903441287

[13] Liu LK, Chou FP, Chen YC, Chyau CC, Ho HH, et al (2009) Effects of Mulberry (Morus alba L.) Extracts on Lipid Homeostasis in Vitro and in Vivo. J Agric Food Chem 57: 7605–7611. 19630385 10.1021/jf9014697

[14] Chen CC, Liu LK, Hsu JD, Huang HP, Yang MY, et al (2005) Mulberry extract inhibits the development of atherosclerosis in cholesterol-fed rabbits. Food Chem 91: 601–607. 10.1021/jf030065w 12926900

[15] Kim SB, Chang BY, Jo YH, Lee SH, Han S-B, et al (2013) Macrophage activating activity of pyrrole alkaloids from Morus alba fruits. J Ethnopharmacol 145: 393–396. 23164765 10.1016/j.jep.2012.11.007

[16] Kang TH, Hur JY, Kim HB, Ryu JH, Kim SY (2006) Neuroprotective effects of the cyanidin-3-O-beta-D-glucopyranoside isolated from mulberry fruit against cerebral ischemia. Neurosci Lett 391: 122–126. 16181734 10.1016/j.neulet.2005.08.053

[17] Kim HG, Ju MS, Shim JS, Kim MC, Lee S-H, et al (2010) Mulberry fruit protects dopaminergic neurons in toxin-induced Parkinson's disease models. Br J Nutr 104: 8–16. 20187987 10.1017/S0007114510000218

[18] Asano N, Yamashita T, Yasuda K, Ikeda K, Kizu H, et al (2001) Polyhydroxylated alkaloids isolated from mulberry trees (Morus alba L.) and silkworms (Bombyx mori L.). J Agric Food Chem 49: 4208–4213. 11559112 10.1021/jf010567e

[19] Wolfe K, Wu X, Liu RH (2003) Antioxidant Activity of Apple Peels. J Agric Food Chem 51: 609–614. 12537430 10.1021/jf020782a

[20] Bettaieb I, Bourgou S, Wannes WA, Hamrouni I, Limam F, et al (2010) Essential Oils, Phenolics, and Antioxidant Activities of Different Parts of Cumin (Cuminum cyminum L.). J Agric Food Chem 58: 10410–10418. 20809647 10.1021/jf102248j

[21] Granica S, Czerwińska ME, Piwowarski JP, Ziaja M, Kiss AK (2013) Chemical Composition, Antioxidative and Anti-Inflammatory Activity of Extracts Prepared from Aerial Parts of Oenothera biennis L. and Oenothera paradoxa Hudziok Obtained after Seeds Cultivation. J Agric Food Chem 61: 801–810. 23311638 10.1021/jf304002h

[22] Valentao P, Fernandes E, Carvalho F, Andrade PB, Seabra RM, et al (2001) Antioxidant Activity of Centaurium erythraea Infusion Evidenced by Its Superoxide Radical Scavenging and Xanthine Oxidase Inhibitory Activity. J Agric Food Chem 49: 3476–3479. 11453794 10.1021/jf001145s

[23] Li T, Zhang X, Song Y, Liu J (2005) A microplate-based screening method for α-glucosidase inhibitors. Chin J Clin Pharm Ther 10: 1129–1131.

[24] Orhan N, Aslan M, Orhan DD, Ergun F, Yesilada E (2006) In-vivo assessment of antidiabetic and antioxidant activities of grapevine leaves (Vitis vinifera) in diabetic rats. J Ethnopharmacol 108: 280–286. 16824713 10.1016/j.jep.2006.05.010

[25] Armbruster DA (1987) Fructosamine: structure, analysis, and clinical usefulness. Clin Chem 33: 2153–2163. 3319287

[26] Bradford MM (1976) A rapid and sensitive method for the quantitation of microgram quantities of protein utilizing the principle of protein-dye binding. Anal Biochem 72: 248–254. 942051 10.1016/0003-2697(76)90527-3

[27] Oyanagui Y (1984) Reevaluation of assay methods and establishment of kit for superoxide dismutase activity. Anal Biochem 142: 290–296. 6099057 10.1016/0003-2697(84)90467-6

[28] Sedlak J, Lindsay RH (1968) Estimation of total, protein-bound, and nonprotein sulfhydryl groups in tissue with Ellman's reagent. Anal Biochem 25: 192–205. 4973948 10.1016/0003-2697(68)90092-4

[29] Hong J-H, Ahn J-M, Choi S-W, Rhee S-J (2004) The effects of mulberry fruit on the antioxidative defense systems and oxidative stress in the erythrocytes of streptozotocin-induced diabetic rats. Nutr Sci 7: 127–132.

[30] Bennini B, Chulia AJ, Kaouadji M, Thomasson F (1992) Phytochemistry of the Ericaceae. Part 2. Flavonoid glycosides from Erica cinera. Phytochemistry 31: 2483–2486.

[31] Merfort I, Wendisch D (1987) Flavonoid glycosides from Arnica montana and Arnica chamissonis. Planta Med 53: 434–437. 17269063 10.1055/s-2006-962766

[32] Wu T, Abdulla R, Yang Y, Aisa HA (2008) Flavonoids from Gossypium hirsutum flowers. Chem Nat Compd 44: 370–371.

[33] El Mousallami AMD, Afifi MS, Hussein SAM (2002) Acylated flavonol diglucosides from Lotus polyphyllos. Phytochemistry 60: 807–811. 12150804 10.1016/s0031-9422(02)00177-2

[34] Kishore PH, Reddy MVB, Gunasekar D, Murthy MM, Caux C, et al (2003) A new coumestan from Tephrosia calophylla. Chem Pharm Bull 51: 194–196. 12576655 10.1248/cpb.51.194

[35] Kim MN, Le Scao-Bogaert F, Paris M (1992) Flavonoids from Carthamus tinctorius flowers. Planta Med 58: 285–286. 17226471 10.1055/s-2006-961460

[36] Pistelli L, Spera K, Flamini G, Mele S, Morelli I (1996) Isoflavonoids and chalcones from Anthyllis hermanniae. Phytochemistry 42: 1455–1458.

[37] Dellemonache G, De Rosa MC, Scurria R, Vitali A, Cuteri A, et al (1995) Cell-suspension cultures of maclura-pomifera. 3. Comparison between metabolite productions in cell-culture and in whole-plant of Maclura-pomifera. Phytochemistry 39: 575–580. 19830921 10.1016/0031-9422(94)00971-u

[38] Yao S, Min Z-D (2005) Two new chalcones from the leaves of Artocarpus heterophyllus. Chin J Nat Med 3: 219–223.

[39] Orhan DD (2003) Novel flavanone glucoside with free radical scavenging properties from Galium fissurense. Pharm Biol 41: 475–478.

[40] Shen Z, Theander O (1985) The constituents of conifer needles. Part 10. Flavonoid glycosides from needles of Pinus massoniana. Phytochemistry 24: 155–158.

[41] Markham KR, Webby RF, Vilain C (1984) 7-O-Methyl-(2R:3R)-dihydroquercetin 5-O-β-D-glucoside and other flavonoids from Podocarpus nivalis. Phytochemistry 23: 2049–2052.

[42] Turner A, Chen S-N, Nikolic D, van Breemen R, Farnsworth NR, et al (2007) Coumaroyl Iridoids and a Depside from Cranberry (Vaccinium macrocarpon). J Nat Prod 70: 253–258. 17269823 10.1021/np060260f PMC1847405

[43] Seyoum A, Asres K, El-Fiky FK (2006) Structure-radical scavenging activity relationships of flavonoids. Phytochemistry 67: 2058–2070. 16919302 10.1016/j.phytochem.2006.07.002

[44] Sroka Z, Cisowski W (2003) Hydrogen peroxide scavenging, antioxidant and anti-radical activity of some phenolic acids. Food Chem Toxicol 41: 753–758. 12738180 10.1016/s0278-6915(02)00329-0

[45] Reis B, Martins M, Barreto B, Milhazes N, Garrido EM, et al (2010) Structure-Property-Activity Relationship of Phenolic Acids and Derivatives. Protocatechuic Acid Alkyl Esters. J Agric Food Chem 58: 6986–6993. 20446740 10.1021/jf100569j

[46] Reynertson KA, Wallace AM, Adachi S, Gil RR, Yang H, et al (2006) Bioactive Depsides and Anthocyanins from Jaboticaba (Myrciaria cauliflora). J Nat Prod 69: 1228–1230. 16933884 10.1021/np0600999

[47] Tadera K, Minami Y, Takamatsu K, Matsuoka T (2006) Inhibition of alpha-glucosidase and alpha-amylase by flavonoids. J Nutr Sci Vitaminol 52: 149–153. 16802696 10.3177/jnsv.52.149

[48] Wang H, Du YJ, Song HC (2010) alpha-Glucosidase and alpha-amylase inhibitory activities of guava leaves. Food Chem 123: 6–13.

[49] Ryu HW, Lee BW, Curtis-Long MJ, Jung S, Ryu YB, et al (2009) Polyphenols from Broussonetia papyrifera Displaying Potent α-Glucosidase Inhibition. J Agric Food Chem 58: 202–208. 10.1021/jf903068k 19954213

[50] Rao RJ, Tiwari AK, Kumar US, Reddy SV, Ali AZ, et al (2003) Novel 3-O-acyl mesquitol analogues as free-radical scavengers and enzyme inhibitors: Synthesis, biological evaluation and structure-activity relationship. Bioorg Med Chem Lett 13: 2777–2780. 12873513 10.1016/s0960-894x(03)00494-3

[51] Pehluvan M, Karlidag H, Turan M (2012) Heavy metal levels of mulberry (Morus alba L.) grown at different distances from the roadsides. J Anim Plant Sci 22: 665–670.

[52] Wurms KV, Cooney JM (2006) Isolation of a new phenolic compound, 3, 5-dihydroxy-2-(methoxycarbonylmethyl)phenyl 3, 4-dihydroxybenzoate, from leaves of Actinidia chinensis (kiwifruit). Asian J Biochem 1: 325–332.

[53] Hillenbrand M, Zapp J, Becker H (2004) Depsides from the petals of Papaver rhoeas. Planta Med 70: 380–382. 15095160 10.1055/s-2004-818956

[54] Lv P-C, Xiong J, Chen J, Wang K-R, Mao W-J, et al (2010) Novel depsides as potential anti-inflammatory agents with potent inhibitory activity against Escherichia coli-induced interleukin-8 production. J Enzyme Inhib Med Chem 25: 590–595. 20235754 10.3109/14756360903357551

